# Feasibility, Acceptability, and Potential Effects of a Digital Oral Anticancer Agent Intervention: Protocol for a Pilot Randomized Controlled Trial

**DOI:** 10.2196/55475

**Published:** 2025-03-26

**Authors:** Saima Ahmed, Christine Maheu, Walter Gotlieb, Gerald Batist, Carmen G Loiselle

**Affiliations:** 1 Division of Experimental Medicine Faculty of Medicine and Health Sciences McGill University Montreal, QC Canada; 2 Segal Cancer Centre Centre intégré universitaire de santé et de services sociaux du Centre-Ouest-de l’Île-de Montréal Montreal, QC Canada; 3 Ingram School of Nursing Faculty of Medicine and Health Sciences McGill University Montreal, QC Canada; 4 Department of Oncology Faculty of Medicine and Health Sciences McGill University Montreal, QC Canada; 5 Department of Obstetrics and Gynecology Faculty of Medicine and Health Sciences McGill University Montreal, QC Canada

**Keywords:** oral anticancer agent, supportive intervention, medication adherence, cancer, oncology, feasibility, acceptability, digital health, anticancer, adherence, compliance, RCT, randomized controlled trial, drug, pharmacy, pharmacology, pharmacotherapy, pharmaceutic, pharmaceutical, medication, mobile phone

## Abstract

**Background:**

Individuals taking oral anticancer agents (OAAs) often face important challenges, requiring more timely informational support, ongoing monitoring, and side effect management.

**Objective:**

This study, guided by the Self-Efficacy Theory, aims to assess the feasibility, acceptability, and potential effects of a comprehensive, digital OAA intervention.

**Methods:**

A 2-arm, mixed methods, pilot randomized controlled trial took place at a large university-affiliated cancer center in Montreal, Quebec, Canada. Participants (N=52) completed baseline self-report e-questionnaires and subsequently were randomly assigned to the experimental group (intervention plus usual care, n=26) or control group (usual care only, n=26). The study intervention, designed to increase medication adherence via medication adherence self-efficacy and decreased symptom distress, included (1) OAA informational videos, (2) OAA-related e-handouts and other supportive resources, (3) nurse-led follow-up calls, and (4) e-reminders to take OAAs. The e-questionnaires were completed once a week for the first month and every 2 weeks for the subsequent 4 months, or until OAA treatment was completed. A subset from both groups (n=20) participated in semistructured interviews once they completed the study requirements. Study feasibility is assessed using recruitment, retention, and response rates, as well as intervention uptake. Through e-questionnaires and exit interviews, intervention acceptability is to be assessed prospectively at baseline and retrospectively upon study completion. Potential effects are then assessed via medication adherence self-efficacy, medication adherence self-report, and symptom distress.

**Results:**

Data collection was completed by December 2023 with a final sample size of 41. Results are expected to be published in 2025.

**Conclusions:**

This study relies on a theoretically based, OAA digital intervention with modalities tailored to the needs and preferences of participants. The use of quantitative and qualitative methods enriches our understanding of the potential contributions of the intervention. In addition, following participants over the course of treatment captures potential changes in oral treatment–related processes and outcomes.

**Trial Registration:**

ClinicalTrials.gov NCT04984850; https://www.clinicaltrials.gov/study/nct04984850

**International Registered Report Identifier (IRRID):**

DERR1-10.2196/55475

## Introduction

### Background

It is estimated that 18.1 million new cancer diagnoses occur globally every year [1,2]. Whereas survival rates vary among cancer diagnoses and countries, mortality rates for the most prevalent cancers in high-income countries continue to decrease [2,3]. Individuals with cancer are living longer and with higher quality of life due to improvements in prevention, detection, and advancements in treatment [4]. More specifically, driven by cost-effectiveness, patient convenience, and the potential for improved patient outcomes, the use of orally administered anticancer drugs continues to grow. It is now estimated that 60% of all new cancer medications currently in development are oral, across all cancer types and stages [5].

Oral anticancer agents (OAAs), having grown in popularity in the past few years, demonstrate equivalent efficacy, safety, and outcomes as intravenous chemotherapy, while being less invasive and easier to administer [6]. As OAAs are taken at home rather than in cancer centers or hospitals, medication management resides with patients- requiring them to be active in their care [7]. For OAAs to be as effective as possible and demonstrate outcomes equivalent to those seen in clinical trials, patients must follow best practices for their treatment, resulting in added responsibilities for medication management [8]. These include attention to treatment adherence, as well as monitoring and management of side effects and adverse events, especially at OAA treatment onset when side effects and toxicity may be high [9,10]. However, the literature to date suggests that patients often report having unmet OAA-related needs, feeling helpless at home, receiving insufficient knowledge and support to manage their treatment, and having suboptimal medication adherence [10-13].

A Canadian survey conducted among individuals treated for cancer in the last 6 months (N=3300), for instance, found that only 62% of individuals on OAAs reported receiving information and guidance on potential side effects and how to manage them, compared to 74% for radiation and 76% for intravenous chemotherapy. In the same sample, only 67% of individuals on OAAs felt their care provider did everything they could to help with side effects, compared to 73% for radiation and 76% for intravenous chemotherapy [14]. Elsewhere, the lack of OAA information and monitoring for side effects were found to be significantly related to fatigue, nausea and vomiting; change of taste; and poorly managed mouth sores [15].

Medication adherence, the extent to which a person’s medication-related “behavior corresponds with agreed-upon recommendations from their health care provider” [16], denotes a collaborative relationship between the health care provider and patient where the patient plays an active role in taking their prescribed treatment [17]. Medication adherence is construed as one of the primary determinants of treatment success, as unwanted alterations in dose and timing affect treatment-related outcomes [18,19]. However, medication adherence rates for OAAs vary significantly, with a systematic review across 63 studies reporting adherence rates ranging from 46% to 100% [20]. Lower OAA adherence is found to be related to decreased treatment effectiveness, increased health care utilization, and increased costs due to more physician visits, higher hospitalization rates, longer hospital stays, and in some cases, decreased survival [21-24].

As OAA development and use expands, medication adherence issues related to OAAs are increasingly of interest to multiple stakeholders, including policy makers, insurance companies, drug makers, health care providers, and researchers [25]. A systematic review of factors influencing adherence to oral anticancer drugs identified three potentially modifiable factors that interventions should address: (1) side effects and toxicities, (2) forgetfulness, and (3) the lack of timely information [26]. The American Society of Clinical Oncology and the Oncology Nursing Society jointly released evidence-based guidelines and OAA management standards. These emphasize patient education at OAA initiation and ongoing monitoring throughout treatment to enable early identification of side effects and toxicities, thus preventing complications [27,28]. Consequently, there is a need for more timely and more accessible patient support for individuals taking OAAs [28].

A comprehensive, personalized, digital OAA intervention was developed based on Bandura’s [29] “Self-Efficacy Theory.” One of the intervention goals is to increase medication adherence by increasing medication adherence self-efficacy (SE) and symptom distress. SE refers to individuals’ beliefs in their own ability to successfully perform a specific task related to specific behavior, for instance, remembering to take medication on time to adhere to treatment, or effectively self-managing fatigue experienced from treatment [29,30]. A systematic review of the relationship between SE and medication adherence found a positive link between these two variables in 59 out of the 66 studies reviewed [31]. Behavior is influenced by the interaction between perceived SE and expectations surrounding the outcome of the behavior; thus, medication adherence is affected by a patient’s belief in their capacity to consistently remember to take medication and the belief that consistently taking the medication, as prescribed, will be an effective treatment to kill cancer cells in their body. Knowledge and self-management skills of disease care can enhance SE through expectations [32]. In support of this, a standardized patient education and follow-up intervention for oral chemotherapy by Tokdemir and Kav [33] successfully increased medication adherence SE after the intervention (66.39 vs 71.04; *P*<.05). Herein, we tested a broader multimodal intervention that went beyond patient education.

### Purpose of Study

The aim of this pilot randomized controlled trial (RCT) was to document the feasibility and acceptability of the experimental intervention. Pilot studies are critically important as a first step to address practical, logistical, and methodological issues that may arise. In addition, this pilot RCT seeks to determine whether study components can be executed and delivered to participants as planned and the intervention’s potential impact on medication adherence SE, adherence, and symptom distress among participants on OAAs.

**Figure 1:**
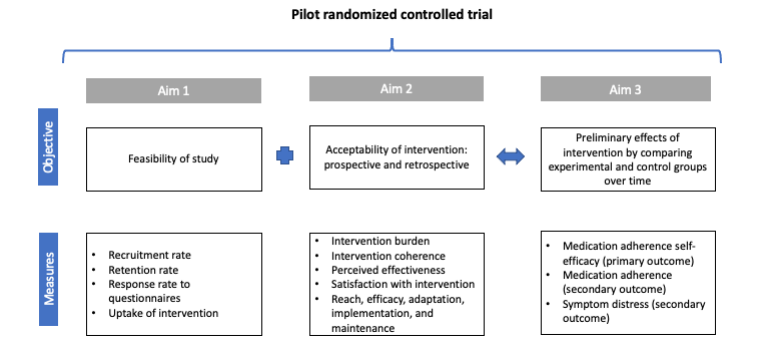
Study aims, objectives, and measures.

More specifically, the study’s first aim was to establish feasibility, defined as “whether the intervention, study design, and procedures can be successfully executed by the researcher and delivered to the participants as planned” [34]. The constructs of feasibility herein include participant recruitment, retention, self-report questionnaire response, and uptake of the intervention. Predetermined objectives and measures of success for each are reviewed in the *Methods* section while Figure 1 provides an overview.

The second aim is to determine the acceptability of the intervention. The definition and measures of acceptability are based upon theoretical framework of acceptability of health care interventions by Sekhon et al [35], which defines acceptability as a multifaceted construct reflecting the appropriateness of the intervention. A key feature of this framework is the distinction between prospective, concurrent, and retrospective acceptability, emphasizing that acceptability can be assessed before, during, and after the intervention as all 3 can have an impact on participant use and access to the intervention. Although Sekhon et al [35] propose 7 concepts of intervention acceptability, only the 3 most relevant are included herein, namely intervention burden, coherence, and perceived effectiveness.

The third aim of the study focuses on documenting the potential effects of the intervention: As stated in the CONSORT (Consolidated Standards of Reporting Trials) on randomized pilot and feasibility trials [36], pilot trials may assess potential effectiveness using surrogate outcomes—substitute measures used as alternatives to clinical outcomes that may be challenging to assess directly [36]. Herein, potential intervention effects are assessed by comparing experimental and control groups over time, from baseline, every 2 weeks (depending on the outcome), and after the intervention. It is hypothesized that over time, compared to the control group, the experimental group will report higher medication adherence SE, higher medication adherence, and lower overall symptom distress.

## Methods

### Design

A prospective, mixed methods, 2-arm, pilot RCT is being conducted to address the study aims and hypothesis.

### Setting

The study takes place at a large academic cancer center in a university-affiliated hospital in Montreal, Quebec, Canada.

### Ethical Considerations

The study received approval from the Psychosocial Research Ethics Committee of CIUSSS West-Central Montreal Research Ethics Board (Project 2021-2861). Participants provided written informed consent. A randomly generated unique number (combination of numbers containing no identifiers) was generated and assigned to each participant such that all data collected were deidentified. As a token of appreciation for the time spent completing study e-questionnaires at baseline and follow-ups, participants received a CAD $10 (a currency exchange rate of CAD $1=US $0.69 is applicable) gift card at baseline as well as an additional one for each set of e-questionnaire completed. In sum, each participant could receive a maximum of CAD $120 in gift cards over the 5-month study period. If they withdrew from the study at any time, they receive a minimum of CAD $10 for the baseline e-questionnaire with an additional CAD $10 for each follow-up e-questionnaire completed.

### Sample

A sample of 52 participants (26 per arm) was to be recruited and randomly assigned, at any moment from the decision to start OAA therapy to the completion of their first oral medication cycle.

#### Sample Size

Sample size calculation was undertaken using procedures provided by the software program G*Power 3 [37]. As per our statistical consultant, the calculation was undertaken to determine adequate power in the determination of the potential effects of the intervention (aim 3), in which a repeated-measures ANOVA with a within-between interaction would be the statistical test used. The parameters for the power calculation included an effect size of 0.25 (standard medium effect size for ANOVA) [38], α of .01, and a power of 0.95. The sample size was further increased to account for a 30% attrition rate over the study duration, determined to be appropriate given a review of attrition rates in supportive oncology trials found a mean of 26% (95% CI 23%-28%) across 18 trials (ie, the original sample size was 36, total with added 30%, attrition is 52) [39].

#### Inclusion Criteria

The inclusion criteria were as follows: being 18 years or older; being seen at the study cancer center; having a diagnosis of cancer at any stage; and being about to start or within the first cycle of oral anticancer treatment (traditional cytotoxic, targeted therapy, or hormonal therapy as adjuvant treatment). Potential participants had to have access to a computer tablet or smartphone device with internet, as well as the ability to communicate, read, and write in English or French.

#### Exclusion Criteria

The exclusion criteria were as follows: receiving intravenous chemotherapy, immunotherapy, or oral hormonal therapy as long-term maintenance treatment for the prevention of cancer’s return or growth of cancer cells after initial treatment, assisting in prolonged remission; any significant physical or cognitive limitations that would prevent the ability to fully participate in the study (as reported by the patient, primary health care provider, or research staff); and being at imminent “end-of-life,” defined as a condition in rapid decline whereby active treatment is stopped and considered in the actual process of dying [40]. We also excluded patients who were already participating in an ongoing clinical trial.

### OAA Experimental Intervention

**Figure 2:**
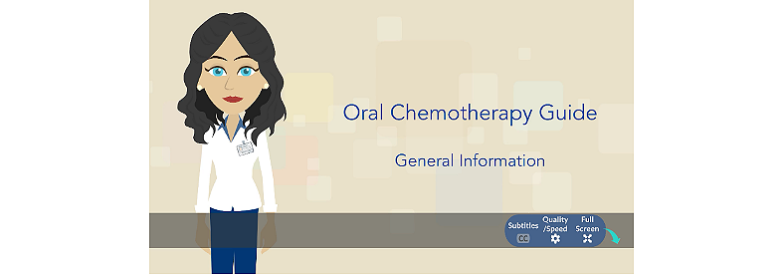
The multimodal OAA intervention contained OAA informational videos on general information (seen here), side effects, support, fertility and work, and symptoms. OAA: oral anticancer agent.

**Figure 3:**
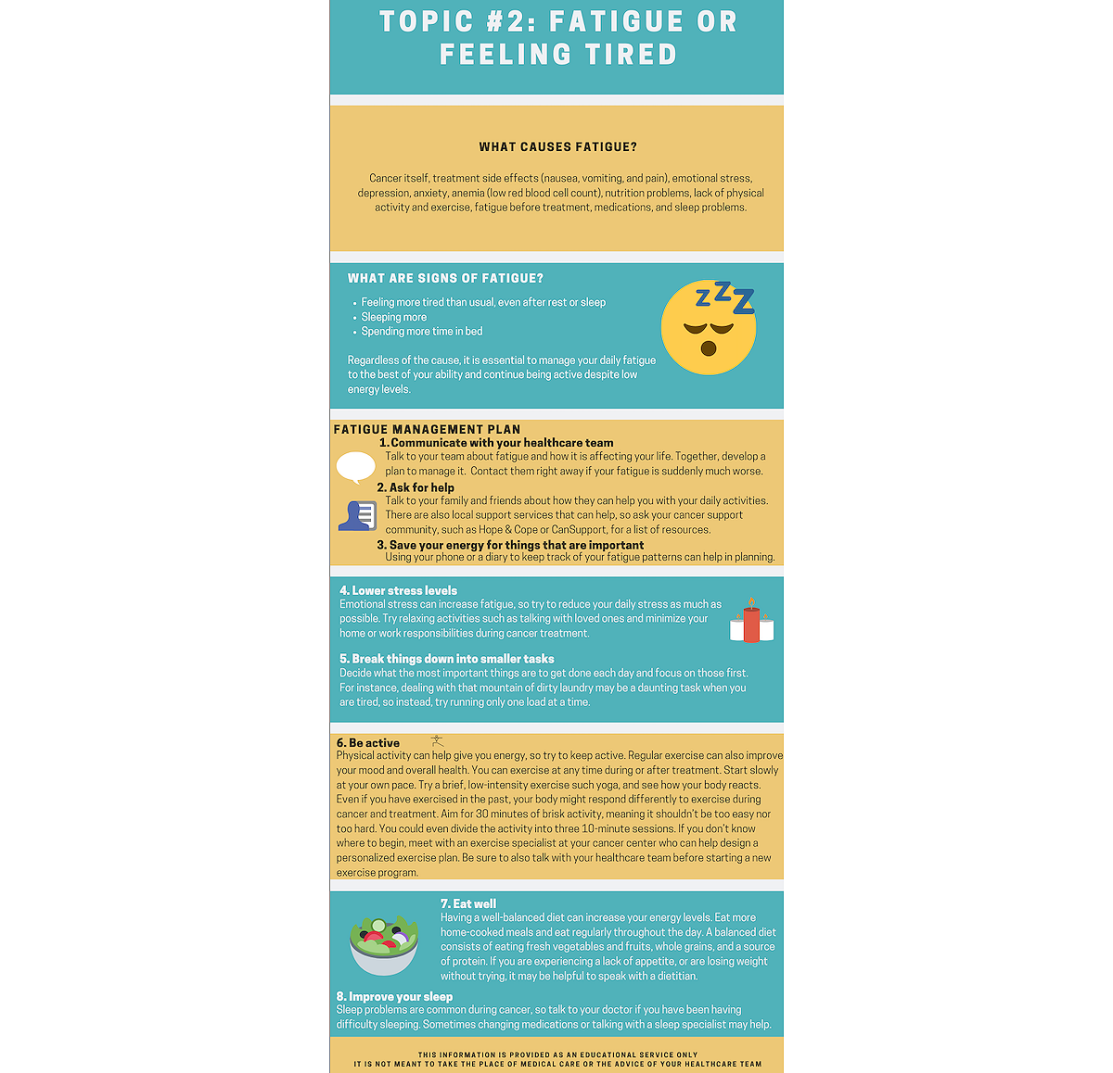
The multimodal OAA intervention contained symptom management tip sheets and additional web-based resources on pain, fatigue (seen here), drowsiness, nausea and vomiting, lack of appetite, shortness of breath, depression, anxiety, well-being, insomnia, fear of cancer recurrence, and work. OAA: oral anticancer agent.

**Figure 4:**
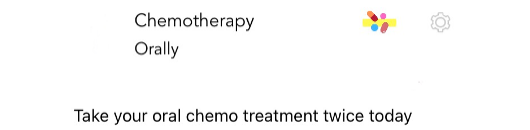
The multimodal OAA intervention contained medication reminders as reminder notification pop-ups. OAA: oral anticancer agent.

All study intervention components were available remotely using a study-specific access code on Belong – Beating Cancer Together [41], a supportive digital platform with a closed community for patients, caregivers, and health care providers at the institution to create networks and connect with other patients [42]. Participants could access the platform on their smartphone or tablet and enter an access code for the study as a closed community in the platform. As opposed to a “one-size-fits-all” approach, the study intervention accords the choice to select the support received. Participants in the experimental group were provided access to all intervention components and chose which specific components to use at any time during study participation. The intervention was developed through rigorous multistakeholder consultation processes, beginning with a comprehensive review of existing OAA-related interventions and evidence by the senior author. Noting no published OAA-specific supportive interventions at the time, the senior author (CGL) secured funding from the Rossy Cancer Network to design and test an OAA intervention, including videos and e-handouts addressing potential side effects and complications related to OAA intake. After meeting with Precare, a company providing educational video resources to patients [43], the first and senior authors gathered initial intervention feedback from oncology nurses, oncologists, researchers, cancer community organizations, patient partners, and informal caregivers or family member representatives. More specifically, these stakeholders provided insights into the content, duration, and overall aspects of the videos and e-handouts, contributing to the refinement of the intervention format and delivery. The final version was thoroughly reviewed by the first and senior author and subsequently integrated into an app [41]. The multimodal OOA intervention components are mentioned in Textbox 1 and Figures 2-4 below.

**Multimodal oral anticancer agent intervention components.**

1. Oral anticancer agent informational videos

- Topics: General information, side effects, support, fertility and work, and symptoms

2. Symptom management tip sheets and additional web-based resources

- Topics: Pain, fatigue, drowsiness, nausea and vomiting, lack of appetite, shortness of breath, depression, anxiety, well-being, insomnia, fear of cancer recurrence, and work

3. Call with a nurse navigator.

- Support and dispatch

4. Medication reminders

- Reminder notification pop-ups

5. Any combination of services above

#### OAA Informational Video

In the context of this study, an evidence-based animated video was developed (Figure 2). The content of the video has been reviewed by multiple stakeholders, including health care providers, patients, and caregivers. The video is available to be watched in English or French, with subtitles available in 16 languages. The video contains 4 parts: general information on oral chemotherapy, side effects, support, fertility and work, and symptoms.

#### Symptom Management Tip Sheets and Additional Web-Based Resources Common Physical and Psychosocial Concerns of Oral Anticancer Therapy

These e-handouts provide knowledge, facts, tips, and additional or telephone resources (Figure 3). The content has been reviewed by multiple stakeholders, including health care providers, patients, and caregivers. The e-handouts are available in French and English on the following 12 topics: pain, fatigue, drowsiness, nausea, lack of appetite, shortness of breath, depression, anxiety, sleep, fear of cancer recurrence, and work. The e-handouts are available for download in PDF format.

#### Follow-up Calls From Oncology Nurse

Participants in the experimental group can receive a call from the oncology nurse specific to their tumor site. A participant may request a phone call at each follow-up e-questionnaire by selecting “I would like to receive a phone call from a nurse” and identifying the topic they would like to discuss. The study coordinator forwards the participant’s name and contact number to the nurse. The participant’s symptom scores from the e-questionnaire are shared with their nurse at this time. The nurse calls the participants and speaks to them on the topic of their choice, and the interaction is documented in the patient chart as a virtual encounter.

#### Medication Reminders

Participants can receive daily e-reminder notifications on their smartphones to take their OAA medication (Figure 4). The e-reminders use preconfigured templates tailored to a 21-day cycle (14 days on per 7 days off) or a 28-day cycle (21 days on and 7 days off) that users must select, with options for once or twice daily reminders. Upon the conclusion of each cycle, users receive a notification prompting them to refill their prescription and reload the 21-day or 28-day cycle template.

In sum, the study intervention was designed to increase SE for OAA adherence through direct mastery experiences (self-management and reminders), vicarious experiences (video), verbal persuasion (phone calls), and feedback (self-management and reminders).

Participants in the control group continued to receive care as usual. This includes follow-up care with their oncologist, contact with their pharmacist and nurse as needed, as well as access to any internal and external supportive services from other health care professionals (eg, psychosocial oncology, social services, physiotherapist, occupational therapist, etc).

### Recruitment, Consent, and Randomization

Participant recruitment occurred at the cancer center in two ways. First, a member of the oncology clinical care team (oncologist, radiation oncologist, nurse, or administrative staff) briefly explained the study and asked patients if they were interested in hearing more about the study. If yes, a member of the study team was informed (in-person, email, or telephone) and contacted the patient. Second, a study poster placed at relevant locations within the cancer center contained the contact information (QR code, email, and telephone number) of the study team. The patients then contacted the team directly.

**Figure 5:**
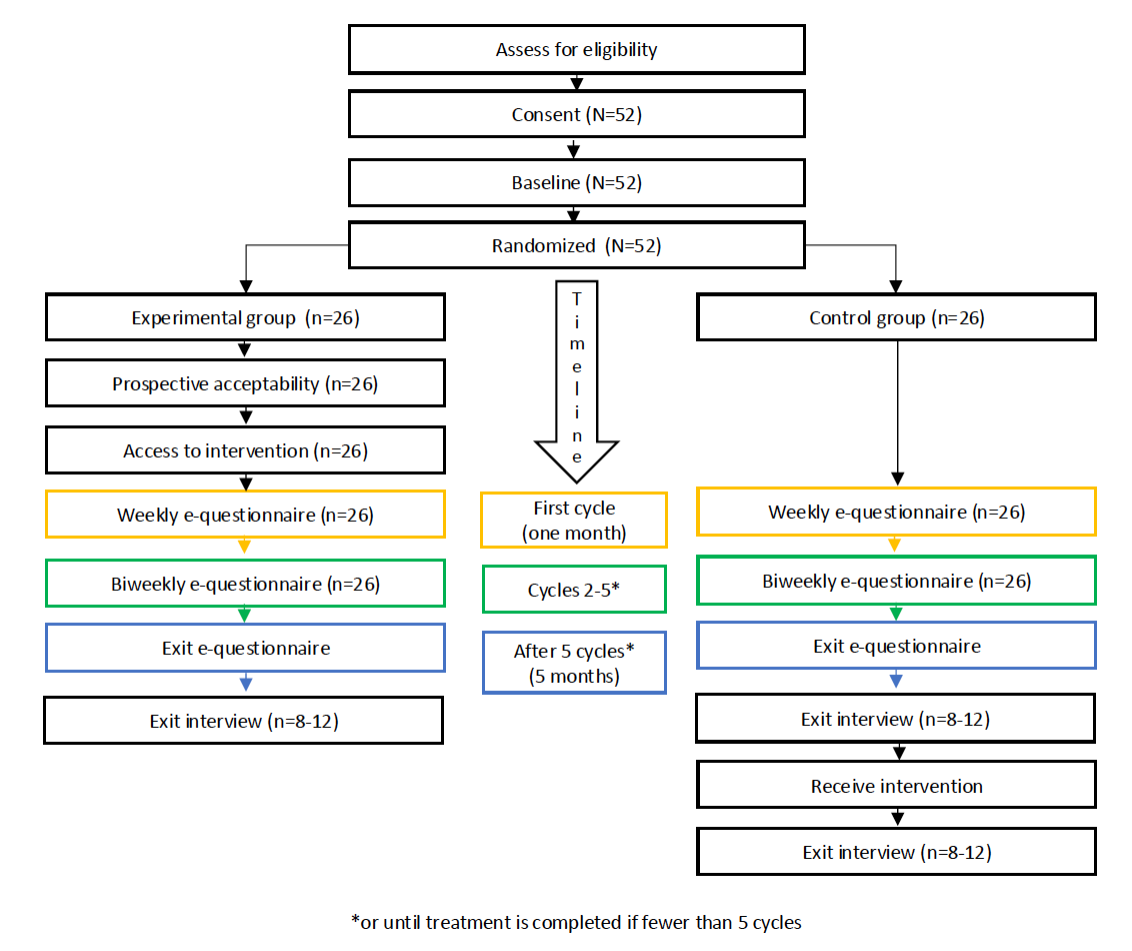
Diagram of study design, measurement points, and timeline.

Interested individuals met with a member of the research team in person or communicated over email or telephone. Study details were provided, and eligibility was verified. If still interested and eligible, a secure link to an electronic consent form was emailed, followed immediately by the baseline questionnaire. Participants were randomly assigned to intervention plus usual care (experimental group, n=26) or usual care only (control group, n=26). Once initial consent was given, those in the experimental group reconsented. Participants in the control group were blinded to group assignment (Article 3.7A of Tri-Council Policy Statement 2) [44]. The randomization sequence was determined on R (R Core Team), a software program, using the randomized R package for clinical trials [45]. Diagram of study design, measurement points, and timeline is shown in Figure 5.

### Data Collection

In both groups, follow-up e-questionnaires were completed every week for the first month and 2 weeks for the following 4 months, or until treatment was completed (if less than 5 months). Given the considerable variability in the duration of time patients may remain on OAAs, the study duration of 5 months was established as a long enough period in consultation with medical oncologists and pharmacists and was deemed appropriate for assessing the primary outcomes of feasibility and acceptability. Participants were monitored more closely during the first treatment cycle, as this period is critical for identifying potential toxicities and making necessary dosage adjustments. Furthermore, it is crucial to establish positive medication adherence behaviors early in the treatment process [46]. After 5 months or until OAA treatment was completed (if less than 5 months), participants completed the exit questionnaire, and a subset of participants in the experimental group (n=10) and control group (n=10) who had completed the study were invited to participate in a semistructured interview.

All e-questionnaires were completed on Qualtrics, a secure web-based electronic data capture system licensed through McGill University [47]. A data management plan between the university and the affiliated hospital was established for the study (Multimedia Appendix 1). Details of each measure and timepoint are provided in Table 1.

Baseline questionnaires completed by all participants included sociodemographic and medical characteristics, cancer information-seeking preferences, and OAA knowledge.

**Table 1:** Study data collection.

Objective and measure			Instrument		Items, n		Time of collection				
						Baseline		Weekly for the first cycle	Every 2 weeks for cycles 2-5	Final questionnaire	Study completion
**Baseline information**											
	Sociodemographics	—^a^		12		✓		—	—	—	—
	Medical characteristics	—		7		✓		—	—	—	—
	Oral anticancer agent knowledge	—		9		✓		—	—	✓	—
	Cancer information-seeking preferences	Cancer information-seeking profiles		1		✓		—	—	✓	—
**Feasibility of study**											
	Recruitment rate	—		—		—		—	—	—	✓
	Retention rate	—		—		—		—	—	—	✓
	Response to questionnaire	—		—		—		—	—	—	✓
	Intervention uptake	—		—		—		—	—	—	✓
**Prospective acceptability of intervention**											
	Intervention burden perceived effectiveness	Acceptability E-scale for web-based patient-reported outcomes in cancer care		3		✓		—	—	—	—
**Retrospective acceptability of intervention**											
	Intervention burden, perceived effectiveness, and intervention coherence	Acceptability E-scale for web-based patient-reported outcomes in cancer care		5		—		—	—	✓	—
	Exit interview	—		—		—		—	—	—	✓
**Preliminary effects of the intervention**											
	Medication adherence	Proportion of days covered		Chart review		—		—	—	—	✓
	Medication adherence	Medication Adherence Rating Scale (Professor Rob Horne)		5		—		✓	✓	—	—
	Medication adherence self-efficacy	Medication Adherence Self-Efficacy Scale		20		✓		✓	✓	—	—
	Symptom distress	Edmonton Assessment Scale revised		12		✓		✓	✓	—	—

^a^Not applicable.

### Measures

#### Sociodemographics and Medical Characteristics

At baseline, participants completed a sociodemographic questionnaire identifying their sex, gender, age, marital status, work status, country of birth, languages spoken, education, and income. They were also asked to complete a medical questionnaire identifying their current diagnosis, cancer stage, coverage of their OAA medication, other medications they are taking on a regular basis, and treatment or treatments received.

#### Cancer Information-Seeking Preferences Scale

This brief, self-report questionnaire based on Self-Evaluation Theory [48] contains 5 statements related to distinct preferences for cancer information. Respondents select the one that best describes how they go about getting information about their cancer: (1) intense—“I seek as much information as possible about my cancer,” (2) complementary—“I seek information about my cancer that adds to what I was told,” (3) peer-focused—“I seek cancer information from others diagnosed with same cancer,” (4) minimal—“I do not seek information about my cancer,” and (5) guarded—“Cancer is stressful enough; I only seek information about my cancer that is hopeful.”

In a large sample (N=2142), participants treated for cancer within the past 6 months responded to the Cancer Information-Seeking Preferences (CISP) scale and patient satisfaction survey (Ambulatory Oncology Patient Satisfaction Survey). A total of 50.2% (1076/2142) selected complementary, 25.2% (539/2142) selected minimal, 14.4% (309/2142) selected guarded, 6.4% (137/2142) selected peer-focused, and 3.8% (81/2142) selected intense, with intense seekers reporting lower satisfaction with cancer care [48,49]. The CISP provides context to the participants’ preferences and uptake of the intervention.

#### Knowledge

The 7-item oral chemotherapy knowledge questionnaire was developed by SA and CGL for the purpose of this study. The scale contains 7 true or false items pertaining to OAA knowledge and self-management (eg, “If I forget to take my oral chemo, I should not double the next dose”).

The study feasibility (aim 1) will be determined by the recruitment rate, retention rate, response rate to e-questionnaires, and uptake of the intervention (Textbox 2).

To assess the acceptability of the intervention (aim 2), intervention burden, intervention coherence, and perceived effectiveness were assessed prospectively at baseline and retrospectively at exit [35] by participants in the experimental group. They were asked to complete the Acceptability E-scale for web-based, patient-reported outcomes in cancer care by Tariman et al [56], requiring a mean score of 80% or higher as the objective. It evaluates the acceptability and usability of computerized health-related programs in oncology. The scale has a reliability of 0.757. It contains 6 items that are rated from 1 (very difficult) to 5 (easy to understand), with total scores ranging from 6 to 30. Table 2 presents constructs and definitions of acceptability as well as baseline and exit questions. In addition, postintervention acceptability was assessed in exit interviews with a subsample of participants (n=20, 10 per group) using an author-generated semistructured interview guide developed based on relevant questions using questions based on the RE-AIM (Reach, Efficacy, Adoption, Implementation, and Maintenance) framework by Glasgow et al [57] to evaluate health behavior interventions (Multimedia Appendix 2). Questions explored participants’ perceptions of OAA information and support, such as “What are your general impressions of the information and support you received during your OAA treatment?” Participant selection for interviews was convenient. Participants were approached in person or over the telephone, and interviews took place in person or over the telephone by the first author, lasting between 30-60 minutes. Only the researchers and participants were present for the interview. After the total number of subsample participants (n=20) had been interviewed, the first author analyzed the interviews to ensure data saturation had been reached. No additional interviews were required.

The potential effects of the intervention (aim 3) will be assessed by comparing experimental and control groups over time, from baseline, every 2 weeks (depending on the outcome), and after the intervention in terms of the following outcomes: medication adherence SE, medication adherence (self-report and pharmacy records), and symptom distress.

**Study feasibility objectives.**

**Recruitment rate**

- Calculated by dividing the total number of participants recruited throughout the study by the number of months recruitment occurred.
- Objective: Based on clinical estimates of eligible individuals, approximately 3 to 4 participants were recruited each month.

**Retention rate**

- Calculated by comparing the number of participants who complete baseline e-questionnaires to the number of participants who complete study exit e-questionnaires.
- Objective: Of participants who begin the study, ≥45% complete the study, and reasons for dropout are documented if participants wish to share [39,50].

**Response rate to study e-questionnaires**

- Determined by the number of completed follow-up e-questionnaire assessments for participants who complete the study.
- Objective: Of participants who complete the study, ≥70% complete outcome measures across all time points. This is slightly higher than the 60% minimum required by biomedical journals [51], typical for web-based questionnaires and patient acceptability and satisfaction research [52,53].

**Uptake of intervention**

- Nature of intervention access (modality, topics, and time points). Uptake of the intervention will be assessed by the number of participants who access the platform, and the number of times each modality was accessed throughout the study duration.
- Objective: Of participants in the experimental group, ≥85% will access at least one intervention modality [54,55].

**Table 2:** Constructs and definitions of acceptability [35] as well as questions asked at baseline and exit [56].

Construct of acceptability	Definition	Question at baseline	Question at exit
Intervention burden	Perceived amount of effort required to participate in the intervention	Do you anticipate the amount of time you will spend reading and watching video or videos in this study will be acceptable?	Was the amount of time you spent reading the information on the e-handouts acceptable? Was the amount of time you spent watching the video or videos acceptable? How easy was it for you to access and use the information and support offered in the study? (overall)
Intervention coherence	Extent to which participant understands the intervention and how it works	—^a^	How understandable was the information in the e-handouts? How understandable was the information in the videos?
Perceived effectiveness	Extent to which the participants perceive the intervention as likely to achieve its purpose	How helpful do you anticipate the information and support offered in this study will be in helping you manage your treatment? (treatment management) How helpful do you anticipate the information and support offered in this study will be in reminding you to take your medication? (reminders)	How helpful was the information and support offered in this study in helping you manage your treatment? (treatment management) How helpful was the information and support offered in this study in reminding you to take your medication? (reminders)

^a^Not applicable.

#### Medication Adherence Self-Efficacy

The Medication Adherence Self-Efficacy Scale (MASES) [58] asks about participants’ level of confidence in taking their medication. The original scale contains 25 items, each rated from 1 (not at all sure) to 3 (extremely sure), with a total score calculated by summing the responses. Initially developed within the context of antihypertensive medication, the scale has been modified and adapted into 24-items for oncology oral agents [33]. For this study, 4 items were removed, and 20 items were used. The 4 items removed were not deemed suitable for the study as they pertain to taking the medication for the rest of their life, coming home late from work, being in a public area, and being afraid of becoming dependent on the medication.

#### Medication Adherence via Proportion of Days Covered

Participants were asked to provide the name and telephone number of their pharmacy, as well as consent for the research team to contact the pharmacy for records to calculate the proportion of days covered (PDC), in order to obtain the average adherence of each participant over 5 cycles of OAAs. PDC is defined as the “sum of the days” supply for all fills of a given drug in a particular time period, divided by the number of days in the time period” [59]. PDC is preferred over the medication possession ratio as the medication possession ratio can overestimate adherence for patients who refill their prescriptions early [59]. PDC will be assessed as the mean PDC for each group.

#### Medication Adherence via the Medication Adherence Report Scale

The Medication Adherence Report Scale (MARS-5; Professor Rob Horne) [60] is a validated measure of medication adherence, with a Cronbach α of 0.67 [60,61]. It is the shorter version of the MARS-10. MARS-5 contains 5 items, each rated from 1 (always) to 5 (never). Total scores range from 5 to 25. In addition, participants are asked specifics about their OAA regimen such as timing, dose delays, interruptions, and stoppages [62].

#### Symptom Distress

Physical and psychosocial distress is measured using the Edmonton Symptom Assessment Scale Revised (ESAS-r). The current version, ESAS-r, has been revised to include psychosocial needs; depression, anxiety, and well-being [63,64]. Each item is rated from 0 (none) to 10 (worst possible). The scale has been tested in cancer populations (Cronbach **α**= 0.71).

### Data Analysis

Statistical analyses to be performed rely on Microsoft Excel, SPSS (version 25; IBM Corp) [65], and R (R Project for Statistical Computing) software [66]. For PDC, independent sample 1-tailed *t* tests will be performed to calculate the difference between 2 independent means of the experimental and control groups at one time point—study completion. Changes over time in MARS-5, MASES, and ESAS-r will be assessed using repeated-measures ANOVA. Between-group analyses will be conducted to examine how each group changed over time, and within-group analyses will be conducted to examine how participants changed over time.

The relationship between objective (PDC) and subjective (MARS-5) measures of medication adherence will be assessed using Spearman correlation coefficients. Oral chemotherapy knowledge at baseline and study exit will be compared using paired sample 1-tailed *t* tests.

Interviews were conducted individually, audio recorded, and transcribed verbatim by the first author, a doctoral candidate at the time with experience in qualitative research in oncology working on her dissertation. A reflexive journal was kept to document thoughts and feelings to recognize, acknowledge, and mitigate the influence of her role as a researcher and as someone with lived experience as an informal caretaker for a parent. Interview data were analyzed by the same author using thematic analysis as described by Braun and Clarke [67,68], beginning with several thorough readings of participant verbatim content to familiarize the researcher with the data and the identification of significant statements relating to the phenomenon under investigation [69]. Next, significant statements were placed into initial categories and organized into broader themes and subthemes. Themes and subthemes were reviewed, redefined, renamed, and explored as needed until no new themes emerged from the data.

## Results

**Figure 6:**
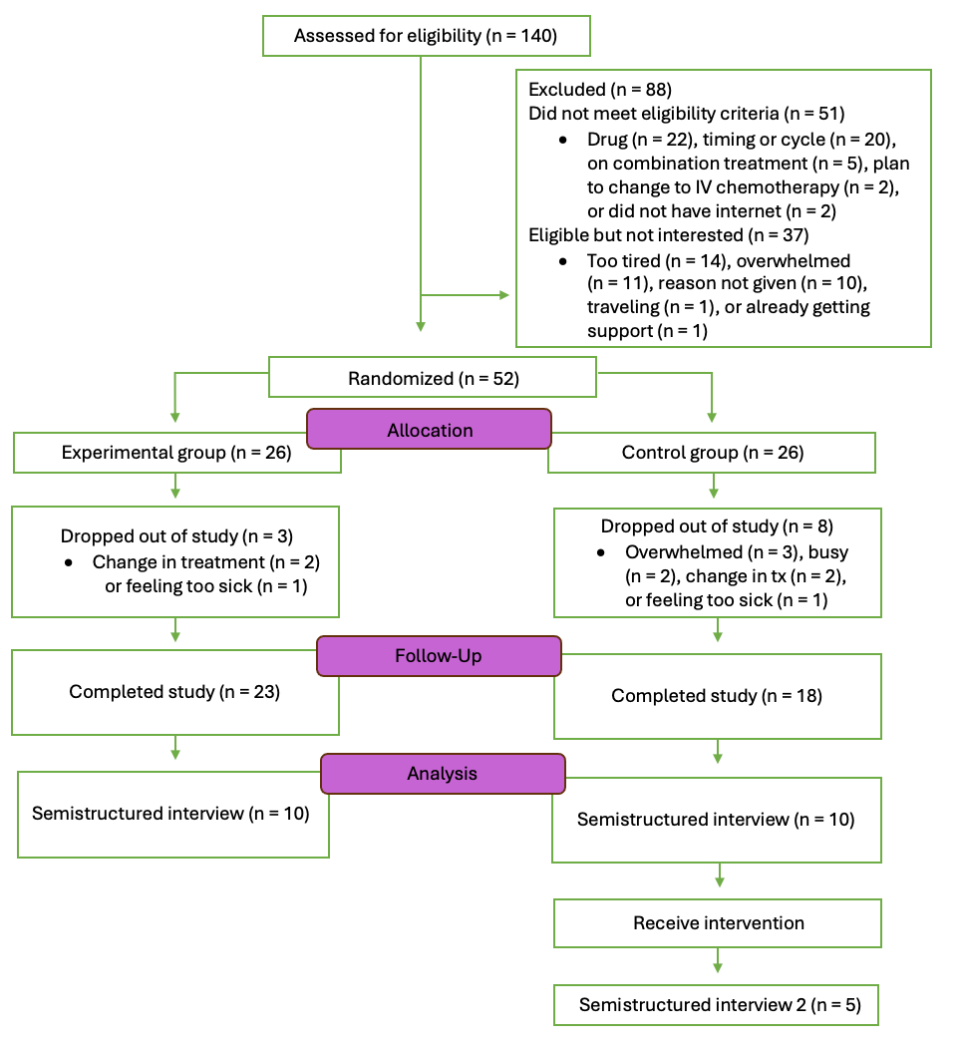
CONSORT (Consolidated Standards of Reporting Trials) flow diagram for the study. IV: intravenous.

Data collection was completed as of December 2023 with a final sample of 41 (experimental group, n=23; control group, n=18), considering 11 dropouts after consent. Results are expected to be published in 2025 in a separate manuscript. Figure 6 presents the CONSORT flow chart for the study.

## Discussion

### Principal Findings

Individuals taking OAAs face many challenges, ultimately impacting medication adherence. While studies have begun to test supportive interventions, there remains a lack of theory-based interventions supported by controlled studies that follow explicit reporting guidelines [70]. This pilot RCT sought to inform the study and intervention feasibility and acceptability from the perspective of patients. Given preliminary insights, it is anticipated that feasibility and acceptability objectives will be achieved. As such, the processes of the study and intervention testing will be successful, and the OAA intervention will be well received by participants. Exit interviews further explore OAA-related experiences and distinct narratives of the intervention, which quantitative measures do not capture. Study results will provide preliminary evidence to assess trends using the potential effects of the comprehensive, theory-based intervention when compared to usual care. The use of qualitative interviews will add further insight to study findings, providing context for the significance or nonsignificance of primary and secondary outcomes. As OAA use continues to grow in upcoming years, the study design and reporting of theory-based intervention will contribute much-needed insights toward how patients on these drugs can best be supported.

Of note, the testing of a remote multimodal intervention was particularly timely amid the COVID-19 pandemic, as oncology teams increasingly performed remote consultations, and patients who are immunocompromised were at higher risk for virus-related complications. Social distancing, isolation, and quarantine all further limited the support and resources available to them.

### Conclusion

As remote consultations are still used for patients who are immunocompromised in the current period, the proposed intervention is still very relevant. Whether the COVID-19 pandemic may have acted as a confounding factor in this study remains unclear. In addition, since the study took place in a single setting, the next steps should include a multisite investigation to determine whether it is scalable and relevant across settings and geographical regions. The study sample is small, with 41 participants completing the study included in the final analysis for preliminary effects. Whereas the final sample is smaller than anticipated (41 vs 52), reliance on mixed methods provides complementary evidence. Dissemination activities related to the study results and its tested intervention include presentations at tumor boards, scientific publications, conference presentations, and diffusion through professional networks and webinars, as well as patient representative groups.

## Appendix

Multimedia Appendix 1 Study data management plan.

Multimedia Appendix 2 Semistructured interview guide.

## Abbreviations

CISP: Cancer Information-Seeking Preferences
CONSORT: Consolidated Standards of Reporting Trials
ESAS-r: Edmonton Symptom Assessment Scale revised
MARS: Medication Adherence Report Scale
MASES: Medication Adherence Self-Efficacy Scale
OAA: oral anticancer agent
PDC: proportion of days covered
RE-AIM: Reach, Efficacy, Adoption, Implementation, and Maintenance
RCT: randomized controlled trial
SE: self-efficacy

## Data Availability

The datasets generated during this study will be available from the corresponding author on reasonable request.
