# Supplementary material for: Feasibility, Acceptability, and Potential Effects of a Digital Oral Anticancer Agent Intervention: Protocol for a Pilot Randomized Controlled Trial
Source: JMIR Res Protoc. 2025 Mar 26;14:e55475. doi: 10.2196/55475 (PMC11982769; doi:10.2196/55475)
Supplement: Multimedia Appendix 1 [file resprot_v14i1e55475_app1.docx]

**DATA MANAGEMENT PLAN**

Version: March 25, 2020

**RESEARCH TEAM**

Dr. Carmen G. Loiselle – Principal Investigator

Dr. Christine Maheu – Co-Investigator

Ms. Saima Ahmed – Graduate student

**QUALTRICS DESCRIPTION**

Qualtrics is a secure, electronic data capture system that supports Health Canada compliance requirements, including user-based privileges, and is FedRamp Authorized (i.e., the gold standard of North American government security compliance). Qualtrics is licensed through McGill University, the Faculty of Medicine and the Faculty of Science Schools and Departments. For this study, the Ingram School of Nursing (at McGill) Qualtrics license will be used. Qualtrics is approved for research use by McGill University and its affiliated research ethics boards. Qualtrics is a powerful, well-supported system that is used by international governments, health care organizations, and academic institutions (e.g., McGill University, Yale University, Healthcare.gov, Microsoft).

**QUALTRICS PLATFORM SECURITY AND PRIVACY FEATURES**

- McGill University’s Qualtrics licenses ensure that data are stored on Canadian servers and comply with Canadian Cloud security privacy laws.
- Qualtrics servers are protected by high-end firewall systems and scans are performed regularly to ensure that any vulnerabilities are quickly found and patched.
- Qualtrics uses Transport Layer Security (TLS) encryption (also known as HTTPS) for all transmitted data.
- Qualtrics survey log-in is password-protected and survey data are automatically backed up daily for recovery purposes only.
- Qualtrics services are hosted by trusted data centers that are independently audited using the industry standard SSAE-18 method.
- Qualtrics has achieved ISO 27001 certification [1]. To independently verify the status of the certification [2].


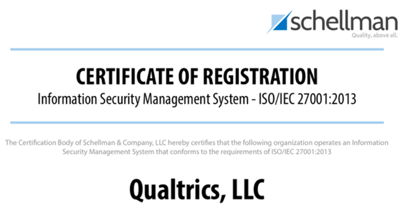


- Federal Risk and Authorization Management Program (FedRAMP) authorization: Qualtrics is FedRamp Authorized. FedRAMP is the standard of U.S. government security compliance, with over 300 controls based on the highly-regarded NIST 800-53 that requires constant monitoring and periodic independent assessments [3].
- The Qualtrics Data Protection Impact Assessment (DPIA) documents Qualtrics’s handling of all data, including personal data.
- A Privacy Impact Assessment (PIA) has been performed and evaluated by an independent third-party assessor.
- HITRUST: To better support healthcare customers, Qualtrics achieved the HITRUST certification in September 2018. The validated report is available upon request.
- General Data Protection Regulation (GDPR) compliant

QUALTRICS SECURITY AND PRIVACY STATEMENTS [4-6]

**STUDY DATA COLLECTION**

Participants will complete electronic consent forms and questionnaires using a unique log-in password (generated by the research team) when logging into Qualtrics to complete study e-questionnaires. Participant consent forms will include an explanation of Qualtrics data collection, including their data privacy and security statements.

**DATA STORAGE AND BACKUP**

Qualtrics data will be securely stored on Canadian servers (this was re-verified via email with the Qualtrics technical support team) [7]

All respondent data are backed up by Qualtrics using two methods: automatic propagation across servers (immediate upon collection) and daily complete off-site encrypted backups. Qualtrics backs up data for disaster recovery purposes only and are not accessed by the third party.

**DATA EXTRACTION, SHARING, AND CODING**

All electronic consent forms, participant contact information, and deidentified data will be stored in Qualtrics. The e-consent forms and participant contact information will be stored in a separate Qualtrics database than the de-identified questionnaire data. The research team will keep a password-protected excel spreadsheet with only the unique ID-participant name link. This password-protected spreadsheet will be stored on the principal investigator’s password-protected home office computer. Normally this link would be stored on a CIUSSS Centre Ouest hospital computer, but the link will need to be stored on a secure, home office computer until the COVID-19 situation has been resolved.

Participant consent forms, contact information, and questionnaire data will not be stored on any portable devices. Only **deidentified** questionnaire data will be extracted from Qualtrics and this will only be done for data analysis purposes. The de-identified database will be securely shared among research team members using McGill University’s OneDrive server [8]

The data will only be used by members of the research team, so a data sharing agreement is unnecessary for these data.

**DATA ANALYSIS**

Dr. Carmen G. Loiselle and a graduate student/statistician will conduct data analyses. The statistical consultant will only have access to the de-identified study data. For more details on the study objectives and the associated data analysis plan, please see the ON-BOARD ethics protocol.

**DATA RETENTION**

The electronic data will be stored for 10 years, after which time the consent forms, participant contact information, and data will be permanently destroyed (deleted) from Qualtrics, the principal investigator’s computer (i.e., the password-protected excel spreadsheet of the ID-participant link), and McGill OneDrive (i.e., the de-identified database). When Qualtrics data are deleted from the platform, all backups of said data are automatically deleted within 90 days.

**MCGILL UNIVERSITY’S FACULTY OF MEDICINE RESEARCH ETHICS OFFICE**

EMAIL COORESPONDENCE

FEBRUARY 5, 2020

McGill University’s Faculty of Medicine Ethics Office has reviewed Qualtrics privacy and data security policies. In their review, the McGill Research Ethics Office reported that:

“Qualtrics is one of the few services that has tools that allow users (e.g. researchers) to better adhere to Quebec’s privacy legislation. For example: when building a survey on the platform or using its cloud services, its Expert Review tool flags information that is deemed sensitive data, provides warning to users/survey responders when sensitive data is being collected and sent, and can redact sensitive information to limit who has access to certain data. The system also includes mechanisms to flag data violations. In terms of Qualtrics’ data privacy policy, it gives users the right to delete personal information and records associated with their accounts. In brief, it is a much more sophisticated platform that has implemented real measures that emphasize and consider data privacy and data security, which is likely why the service comes at a cost to researchers who want to use it.

Additional things we noted: Qualtrics does not allow third-party access to user data (including aggregate data and survey data) which is otherwise shared on many other platforms such as Survey Monkey – I should note that Survey Monkey includes clauses in its policies that allow it to access data from individual survey responses. Qualtrics has implemented technology and services to be GDPR compliant, and allows users to implement privacy tools to prohibit the collection of device identification (e.g., does not collect IP addresses and does not store cookies or trackers on survey respondent devices.)… When researchers can afford the services, I recommend Qualtrics.”

- Sacha Young, McGill University Faculty of Medicine Ethics Review Administrator

References

1. <https://cert.schellmanco.com/?certhash=f4EjsRoh8OCD>
2. [https://www.schellman.com/certificate-directory](about:blank)
3. [https://www.fedramp.gov](about:blank)
4. [https://www.qualtrics.com/platform/security/](about:blank)
5. [https://www.qualtrics.com/security-statement/](about:blank)
6. [https://www.qualtrics.com/privacy-statement/](about:blank)
7. [https://www.qualtrics.com/platform/security/data-sovereignty/](about:blank)
8. [http://kb.mcgill.ca/kb/?ArticleId=5609&source=article&c=12&cid=2#tab:homeTab:crumb:8:artId:5609:src:article](about:blank#tab:homeTab:crumb:8:artId:5609:src:article)
