# Supplementary material for: Feasibility, Acceptability, and Potential Effects of a Digital Oral Anticancer Agent Intervention: Protocol for a Pilot Randomized Controlled Trial
Source: JMIR Res Protoc. 2025 Mar 26;14:e55475. doi: 10.2196/55475 (PMC11982769; doi:10.2196/55475)
Supplement: Multimedia Appendix 2 [file resprot_v14i1e55475_app2.docx]

### Semi-Structured Interview Guide

**Experimental:**

1. Overall, what are your general impressions of the oral chemotherapy information and support you received during the study – this includes the videos, e-handouts, and phone calls?

2. Tell me about the positives, what did you like about the information and support you received? Think back to specifics.

3. Tell me about the negatives, what did you not like?

4. In terms of the videos and e-handouts, what did you think of the information they provided?

 Was it useful or helpful to you? Do you think other individuals on oral chemotherapy (or even their families) would use them and/or find them helpful?

5. Was the information provided easy for you to understand? Are there topics you would have liked to know more about? Are there other changes that you would like to see? In terms of content or even the look of the videos or e-handouts?

6. Did you request a call from a nurse in oncology? If yes, were you satisfied with the help you received?

7. In the long term, would you like to be able to request phone calls from a nurse in oncology when you need them? During later stages of your treatment? Do you think others on oral chemo would find these phone calls useful or helpful?

8. Between the different options available you (videos, e-handouts, and phone calls) is there one that stood out?

9. What did you – yourself- get from the study? Did you feel more supported or better informed? Would you recommend the program (videos, e-handouts, and phone calls) to other individuals on oral chemo? Do you think it would positively or negatively affect their experience taking the medication?

9. Keeping in mind everything we discussed today, on a scale of 1-10, 1 being extremely poor and 10 being excellent, how would you rate the videos, e-handouts, phone calls individually and then everything as a whole?

10. What would make it a 10 (if it is not)?

11. Is there anything else you would like to add to our discussion today?

Other suggestions? Questions?

**Control:**

1. Overall, what are your general impressions of the information and support you received during your oral chemotherapy treatment?

2. Tell me about the positives, what did you like about the information and support you received? Think back to specifics.

3. Tell me about the negatives, what did you not like?

4. What did you think of the information you received? Was it useful or helpful to you?

5. Are there topics you would have liked to know more about?

6. In the long term, what type of support would you like to receive for your treatment? During later stages of your treatment? What type of support do you think others on oral chemo would find useful or helpful?

7. What do you think it would have helped positively affect your experience taking oral chemotherapy medication?

8. Keeping in mind everything we discussed today, on a scale of 1-10, 1 being extremely poor and 10 being excellent, how would you rate the level of support you received for your treatment?

9. What would make it a 10 (if it is not)?

10. Is there anything else you would like to add to our discussion today?

Other suggestions? Questions?
